# Influence of genetic ancestry and socioeconomic status on type 2 diabetes in the diverse Colombian populations of Chocó and Antioquia

**DOI:** 10.1038/s41598-017-17380-4

**Published:** 2017-12-07

**Authors:** Aroon T. Chande, Jessica Rowell, Lavanya Rishishwar, Andrew B. Conley, Emily T. Norris, Augusto Valderrama-Aguirre, Miguel A. Medina-Rivas, I. King Jordan

**Affiliations:** 1 School of Biological Sciences, Georgia Institute of Technology, Atlanta, Georgia USA; 2 IHRC-Georgia Tech Applied Bioinformatics Laboratory, Atlanta, Georgia USA; 3 PanAmerican Bioinformatics Institute, Cali, Valle del Cauca Colombia; 4 Biomedical Research Institute, Faculty of Health, Universidad Libre-Seccional Cali, Cali, Valle del Cauca Colombia; 5 Centro de Investigación en Biodiversidad y Hábitat, Universidad Tecnológica del Chocó, Quibdó, Chocó Colombia

## Abstract

Differences in genetic ancestry and socioeconomic status (SES) among Latin American populations have been linked to health disparities for a number of complex diseases, such as diabetes. We used a population genomic approach to investigate the role that genetic ancestry and socioeconomic status (SES) play in the epidemiology of type 2 diabetes (T2D) for two Colombian populations: Chocó (Afro-Latino) and Antioquia (Mestizo). Chocó has significantly higher predicted genetic risk for T2D compared to Antioquia, and the elevated predicted risk for T2D in Chocó is correlated with higher African ancestry. Despite its elevated predicted genetic risk, the population of Chocó has a three-times lower observed T2D prevalence than Antioquia, indicating that environmental factors better explain differences in T2D outcomes for Colombia. Chocó has substantially lower SES than Antioquia, suggesting that low SES in Chocó serves as a protective factor against T2D. The combination of lower prevalence of T2D and lower SES in Chocó may seem surprising given the protective nature of elevated SES in many populations in developed countries. However, low SES has also been documented to be a protective factor in rural populations in less developed countries, and this appears to be the case when comparing Chocó to Antioquia.

## Introduction

With ongoing economic development, and the lifestyle changes that accompany increased standards of living, the primary disease burden in Latin America is shifting from infectious to non-communicable, complex diseases^1^. In fact, complex common diseases such as heart disease, cancer and diabetes already account for the majority of the morbidity and mortality in the region^2^. Complex multifactorial diseases of this kind are associated with the effects of multiple genetic loci combined with a variety of environmental factors, such as diet, lifestyle and exposure to toxins. The burden of complex disease is not evenly distributed within or between countries in Latin America; genetic and environmental differences among Latino populations often lead to pronounced health disparities^3^. Furthermore, population health disparities in Latin America tend to have a disproportionate impact on vulnerable Native American and Afro-Latino communities^4^.

Diabetes mellitus is a complex multifactorial disease characterized by both a very high disease burden and strikingly disparate impacts among distinct populations in the Americas. For example, type 2 diabetes (T2D) has substantially higher prevalence in both Native Americans and African-Americans compared to European-Americans in the United States (US)^5–10^. The higher prevalence of T2D in these populations has been associated with both genetic and environmental factors. Genetic risk for T2D is correlated with both increased Native American and African ancestry^11–14^, and low socioeconomic status (SES) has also been widely associated with increased T2D prevalence in Native American and African-American populations^15–17^.

Latin American populations are characterized by substantial genetic admixture – with predominant ancestry contributions from Europe, the Americas and Africa – owing to historical patterns of migration, conquest and slavery^18^. Colombia has among the highest levels of three-way genetic admixture seen for any Latin American country^19,20^ and is home to a large population of Afro-descendants^21–23^. Estimates for the size of the Afro-Colombian population range from 9–20 million, making it the second largest population of its kind in Latin America after Brazil. The collaborative ChocoGen research project was conceived to study the genetic heritage of the Afro-Colombian population from the administrative department (*i*.*e*., state) of Chocó, located along Colombia’s Pacific coast (http://www.chocogen.com)^21,22^. The ChocoGen project has the joint aims of (1) characterizing the genetic ancestry of the population of Chocó, and (2) exploring the relationship between genetic ancestry and determinants of health and disease in the region.

The objective of this study was to evaluate the contributions of genetic ancestry and environmental factors to population health disparities in Chocó, and we addressed this issue here via a population genomic analysis of the genetic risk and the observed prevalence of T2D. Our efforts towards this end involve a comparison between the populations of Chocó and the neighboring state of Antioquia, which borders Chocó to the east (Supplementary Figure 1). Despite their proximity, Chocó and Antioquia have very distinct demographic and economic profiles. According the 2005 Colombian census, the population of Chocó was 82% Afro-Colombian, 13% Native American and 5% European/Mestizo, whereas Antioquia was 93% European/Mestizo, 7% Afro-Colombian and ~0.1% Native American^24^. The population of Chocó is considered to be particularly vulnerable, with high levels of poverty and low measures of economic development across a number of indices compared to Antioquia. We chose to focus our comparative study of genetic and health differences between Chocó and Antioquia on T2D for several reasons: (1) its high disease burden, (2) its known contribution to population health disparities, and (3) the relative wealth of knowledge regarding its underlying genetic architecture. We set out to assess whether and how genetic and environmental differences between these two very distinct regions may manifest themselves with respect to population-specific levels of T2D genetic risk and/or differences in observed prevalence for the disease.

## Methods

### Genome sequence and genotype data sources

Whole genome sequence data and whole genome genotype data were analyzed in order to infer the genetic ancestry and admixture profiles for the Colombian populations of Chocó and Antioquia (Table 1). Whole genome genotypes for 94 individuals from Chocó were characterized as part of the ChocoGen research project (http://www.chocogen.com/) as previously described^22^. Sample donors from the ChocoGen project signed informed consent documents indicating their understanding of the potential risks of the project along with how their data would be handled and how their identity would be protected. Collection, genotyping and comparative analyses of human DNA samples were conducted with the approval of the ethics committee of the Universidad Tecnológica del Chocó^22^. All methods were performed in accordance with the journal’s relevant guidelines on the use of human participants. Names and other HIPAA identifiers are removed from all sections of the manuscript, including the supplemental information. No other information that could lead to the identification of study participants is provided.

**Table 1:** Human populations analyzed in this study.

Dataset^1^	Population Sample Name	Short Name	*n* ^2^
ChocoGen^22^	Chocoano in Quibdó, Colombia	Chocó	94
1KGP^25^	Colombian in Medellin, Colombia	Antioquia	94
Reich *et al*.^26^	Embera in Colombia	Embera	5
Reich *et al*.	Quechua in Peru	Quechua	40
Reich *et al*.	Zapotec in Mexico	Zapotec	43
1KGP	Yoruba in Ibadan, Nigeria	Nigeria (Yoruba)	108
1KGP	Iberian populations in Spain	Spain	107
1KGP	Utah residents with NW European ancestry	European-American	99
1KGP	African Ancestry in Southwest US	African-American	61
1KGP	Peruvian in Lima, Peru	Peru	85
1KGP	Finnish in Finland	Finnish	99
1KGP	British in England and Scotland	British	91
1KGP	Toscani in Italy	Italian	107
1KGP	African Caribbean in Barbados	African Caribbean	96
1KGP	Esan in Nigeria	Nigerian (Esan)	99
1KGP	Gambian in Western Division, The Gambia	Gambian	113
1KGP	Luhya in Webuye, Kenya	Keryan	99
1KGP	Mende in Sierra Leone	Sierra Leonean	85

^1^Source of the genome sequence or genotype datasets used in this study. 1KGP refers to the 1000 Genomes Project phase 3 data release^25^.

^2^Number of individuals analyzed for each population.

All of the other data used for the analysis described here corresponds to publicly available and de-identified genome sequences or genotypes. Publicly available whole genome sequences for 94 individuals from Medellín, Antioquia were characterized as part of the 1000 Genomes Project (1KGP)^25^. Whole genome sequences from several additional admixed American populations were taken from the 1KGP for analysis: Utah residents with European ancestry (*n* = 99), African ancestry individuals from the Southwest US (*n* = 61), and a Peruvian population from Lima, Peru (*n* = 85).

Genome sequence and genotype data were also sampled from putative ancestral populations corresponding to the three major continental regions that are known to contribute to genetic admixture in Colombia^18–20,23^: Africa, Europe and the Americas. African ancestry was inferred using whole genome sequences for a Yoruba population from Ibadan, Nigeria (*n* = 108), and European ancestry was inferred using whole genome sequences for an Iberian population from Spain (*n* = 107), both of which were characterized as part of the 1KGP. Whole genome genotypes for three Native American populations – Embera from Colombia (*n* = 5), Quecha from Peru (*n* = 40) and Zapotec from Mexico (*n* = 43) – were taken from a dataset collected as part of a previous study on Native American genetic ancestry^26^.

### Genetic ancestry and admixture analysis

**Figure 1:**
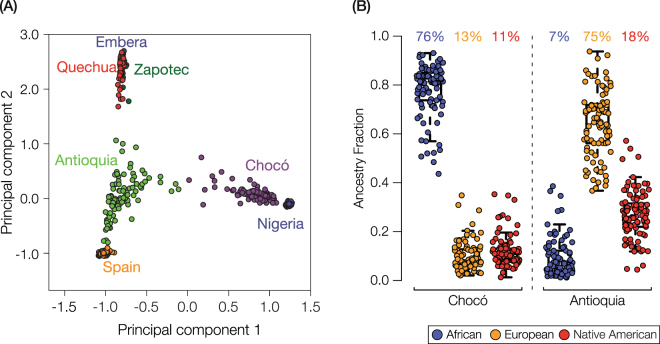
Genetic ancestry of the individuals from Chocó and Antioquia analyzed here. (**A**) Principal components analysis (PCA) plot representing the pairwise distances among individual genomes from the admixed Colombian populations of Chocó and Antioquia along with putative ancestral source populations from Africa (Nigeria), Europe (Spain) and the Americas (Embera, Quechua and Zapotec). (**B**) Box-plot distributions of the ancestry fractions for individuals from Chocó and Antioquia. The population-average values of African (blue), European (orange), and Native American (red) ancestry are shown above the distributions.

Whole genome sequence data and whole genome genotype data were merged using the program PLINK^27^, and the resulting merged single nucleotide polymorphism (SNP) dataset was pruned in order to remove SNPs that are in linkage disequilibrium (*r ≥ *0.05). This resulted in a final dataset of 220,724 SNPs across 736 individual genome samples. Pairwise genomic distances between individuals were calculated as allele sharing distances between all pairs of merged/pruned SNP sets, also using PLINK. The pairwise allele sharing distance matrix was reduced to two-dimensions with principal component analysis (PCA) using the prcomp function from the R package for statistical computing^28^ (Fig. 1A). Ancestry fractions – African, European and Native American – were calculated for each individual genome from Chocó and Antioquia using the program ADMIXTURE^29^, with global reference populations (Table 1) and K = 3 clusters corresponding to each of the major continental ancestry groups (Supplementary Figure 2 and Fig. 1B).

### Type 2 diabetes genetic risk calculation

The underlying genetic architecture of type 2 diabetes (T2D) was assayed from a series of 29 case-control genome-wide association studies (GWAS). T2D SNP association data from these studies were taken from the NHGRI-EBI GWAS catalog^30^. Individual SNP entries from the GWAS catalog were considered to be significantly associated with T2D if (1) the SNP association was uncovered via a case-control study based on at least 100,000 genotyped SNPs, (2) the SNP had the strongest association seen for its genomic locus, and (3) the SNP showed a genome-wide T2D association *P*-value < 1.0e^−5^. This yielded a set of 165 T2D-associated SNPs, and for each SNP the identity of the risk allele (*i*.*e*., specific nucleotide variant) linked to T2D was taken from the study where it was reported.

Imputation was performed on whole genome genotype data from the ChocoGen project in order to facilitate direct comparison of genome-wide T2D risk scores computed for datasets from Chocó (genotypes) and Antioquia (genome sequences). Prior to imputation, the whole genome genotypes of individuals from Chocó were phased using the program SHAPEIT^31,32^ using the 1KGP phase 3 haplotype reference panel. The phased whole genome genotypes from Chocó, consisting of 522,458 SNPs per individual, were then imputed using the program IMPUTE2^33–35^ with the 1KGP phase 3 haplotype reference panel^32^. This process resulted in the imputation of 35,056,488 additional SNPs across all samples. The accuracy of the imputation process was evaluated by comparing the genetic ancestry relationships between individuals from Chocó, computed before and after imputation, and a panel of global reference populations. The observed genetic ancestry relationships for the individuals from Chocó are virtually identical before and after imputation, in support of the accuracy of the imputation process (Supplementary Figure 3).

**Figure 2:**
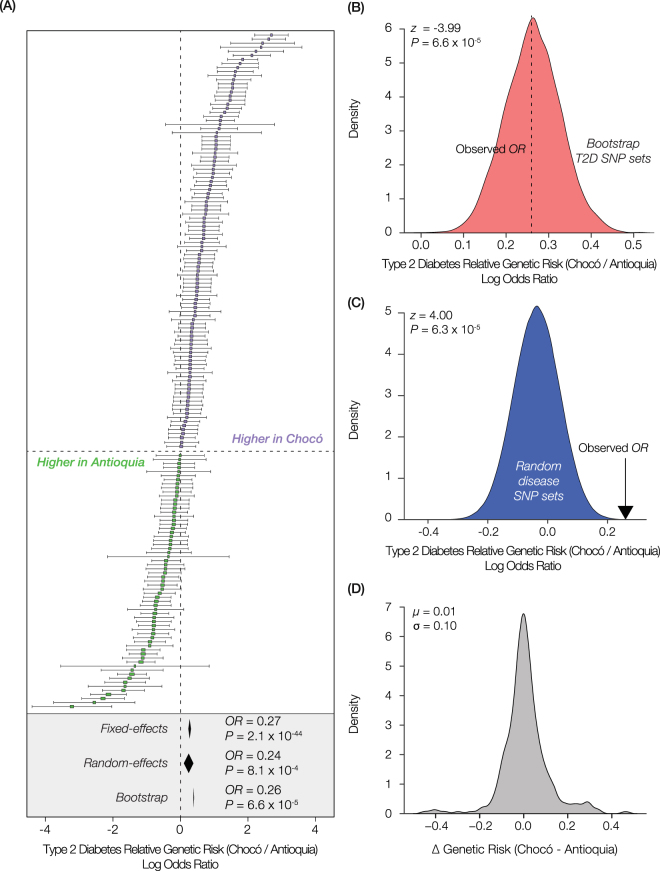
Relative genetic risk for type 2 diabetes (T2D) and genetic ancestry in Chocó versus Antioquia. (**A**) The relative genetic risk of T2D in the two Colombian populations is shown as log odds ratios (*OR*) – Chocó/Antioquia – of the risk versus non-risk allele frequencies for 165 T2D-associated SNPs. The formula for calculating *OR* values is shown in the Methods subsection ‘Type 2 diabetes genetic risk calculation’ (formula 1). *OR* values > 0 indicate greater risk in Chocó (purple), whereas *OR* values < 0 show greater risk in Antioquia (green). 95% confidence intervals (CI) for individual SNP *OR* values are shown. The diamonds below the plot show *OR* values (±95% CI) corresponding to fixed- and random-effects meta-analysis of all 165 T2D-associated SNPs as well as the mean *OR* value from the bootstrap analysis; *P*-values indicating the statistical significance level of the three meta *OR* values are shown. (**B**) The observed *OR* value for the relative genetic risk of T2D (Chocó/Antioquia) is compared to a bootstrap distribution of *OR* values based on random sampling with replacement from the set of T2D-associated SNPs. The values of *z* and *P* for a z-test comparing the distribution of bootstrap T2D SNP *OR* values to 0 are shown. (**C**) The observed *OR* value for the relative genetic risk of T2D (Chocó/Antioquia) is compared to a null distribution of expected *OR* values for randomly simulated SNP sets of the same size as the T2D-associated SNP set. The values of *z* and *P* for a z-test comparing the observed and expected T2D SNP *OR* values are shown. (**D**) The distribution of genetic risk score (*PRS*) differences (Chocó-Antioquia) for 324 diseases is shown along with the mean and standard deviation values for the distribution.

For each T2D-associated SNP, a log odds ratio (*OR*) was used to compute the relative genetic risk of T2D for Chocó compared to Antioquia (Fig. 2A):

1 $$OR=Ln\frac{R{A}_{CHO}/NR{A}_{CHO}}{R{A}_{ANT}/NR{A}_{ANT}}$$

where *RA*_*i*_ and *NRA*_*i*_ are the risk allele frequency and non-risk allele frequency, respectively, in population *i* (*CHO* = Chocó and *ANT* = Antioquia). A meta-analysis was conducted to evaluate the joint effect of all 165 T2D-associated SNPs on the relative genetic risk of T2D in Chocó versus Antioquia using the metafor package in R^36^. 95% confidence intervals for the individual SNP and meta-analysis *OR* values were computed using both fixed- and random-effects models. The fixed- and random-effects models were both computed with moderators via linear (mixed-effects) models.

**Figure 3:**
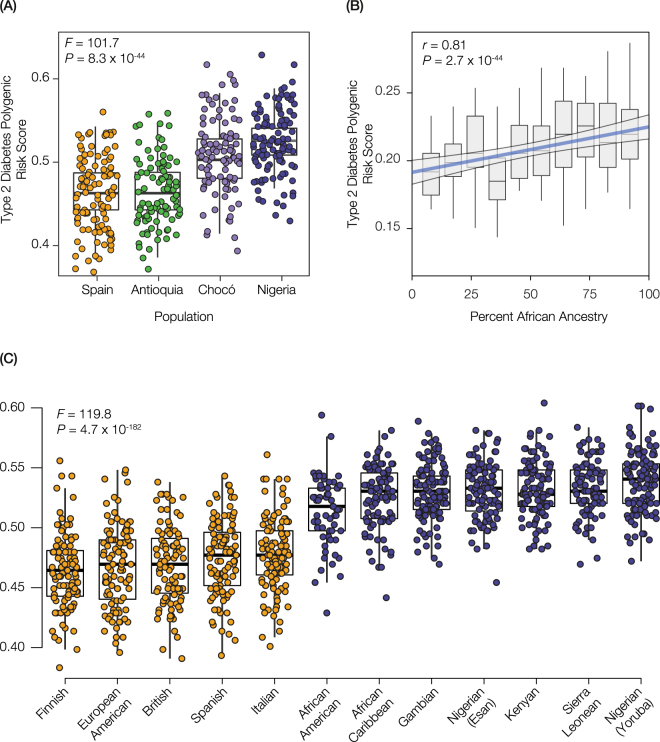
Genetic ancestry and predicted risk for T2D. (**A**) Box-plot distributions of individuals’ T2D polygenic risk scores are shown for four populations: Spain (orange), Antioquia (green), Chocó (purple), and Nigeria (blue). The values of *F* and *P* for an ANOVA test comparing the mean values of the distributions are shown. (**B**) Regression of T2D polygenic risk scores (y-axis) against the percent African ancestry for genome sequences from Colombia and the US (x-axis). Box plots are shown for decile bins, and the linear trend line is shown in blue with 95% CI in gray. The values of *r* and *P* for the Pearson correlation coefficient of the regression are shown. (**C**) Box-plot distributions of individuals’ T2D polygenic risk scores are shown for five European populations (orange) and seven African populations (blue). The values of *F* and *P* for an ANOVA test comparing the mean values of the distributions are shown.

T2D polygenic risk scores (*PRS*) for individual genomes were computed as the unweighted, normalized sum of the number of risk alleles for all 165 T2D-associated SNPs (Fig. 3):

2 $$PRS=\frac{{\sum }_{i=1}^{165}R{A}_{i}}{{\sum }_{i=1}^{165}{A}_{i}}$$

where $R{A}_{i}\,\in \,\{0\,,\,1,\,2\}$, corresponding to homozygous absent, heterozygous or homozygous present risk alleles at each SNP and ${A}_{i}\,\in \,\{0\,,\,1,\,2\}$ corresponding to total number of alleles with basecalls at each SNP. SNP association effect sizes were not used to weight the T2D *PRS* values owing to the fact that the T2D associated SNPs analyzed here were taken from different studies, and the effect size values among studies are not directly comparable.

### Genetic risk calculation controls

A series of controls was performed to check for systematic biases in the frequencies allelic variants used to compare genetic risk scores between populations. (1) Bootstrap: random sampling with replacement from the 165 T2D-associated SNPs was used to create 10,000 replicate SNP sets, each of which was used for genetic risk *OR* calculation and meta-analysis as described above. The resulting distribution of bootstrap meta-analysis *OR* values was compared to the observed value for the T2D SNP set to evaluate how outliers may affect T2D genetic risk calculation and comparison between populations (Fig. 2B). (2) Random disease-associated SNP sets: random sampling of T2D size matched (*n* = 165) disease-associated SNP sets from the NHGRI-EBI GWAS catalog was used to create 500,000 replicate SNP sets, each of which was used for genetic risk *OR* calculation and meta-analysis as described above. The resulting distribution of random disease-associated SNP set meta-analysis *OR* values was compared to the observed value to evaluate whether systematic biases in disease-associated allele frequencies between populations may affect the comparison of genetic risk (Figure 2C). (3) Disease genetic risk comparisons: SNP disease-associations from the NHGRI-EBI GWAS catalog were mined to compare polygenic risk scores (*PRS*), as described above for T2D, for 324 diseases between Chocó and Antioquia in order to assess whether there is any systematic bias in disease genetic risk score computation between the two populations (Fig. 2D).

### Diabetes prevalence and socioeconomic status (SES) data sources

**Figure 4:**
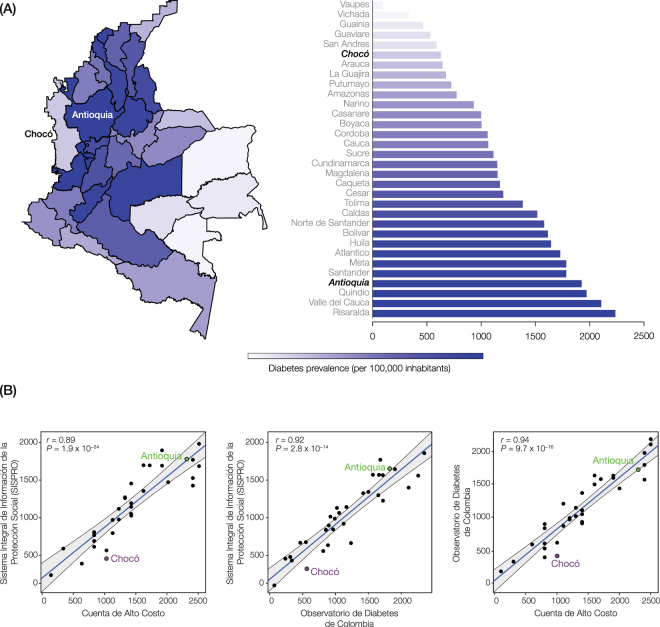
Prevalence of diabetes in Colombia. (**A**) Age-adjusted diabetes mellitus prevalence per 100,000 inhabitants are shown for the 32 Colombian administrative departments (*i*.*e*., states). Diabetes prevalence estimates were averaged across three different epidemiological databases: (1) Cuenta de Alto Costo, (2) Observatorio de Diabetes de Colombia, and (3) the Sistema Integral de Información de la Protección Social. The map was created using the R maptools package^65^ with mapping data from OpenStreetMaps. The cartography in the OpenStreetMap map tiles is licensed under CC BY-SA (www.openstreetmap.org/copyright). The map data are available under the Open Database License © OpenStreetMap contributors. The license terms can be found on the following link: http://creativecommons.org/licenses/by-sa/2.0/. (**B**) Comparison of Colombian diabetes state-by-state prevalence estimates taken from the three different database sources. Regression plots for all three possible pairwise comparisons between the different databases are shown, with the values for Chocó and Antioquia indicated. For each regression, the Pearson correlation *r*-value is shown along with the *P*-value significance level.

Data on age-adjusted diabetes prevalence per 100,000 inhabitants for the Colombian administrative departments (*i*.*e*., states) was taken from three database sources: (1) Cuenta de Alto Costo (https://cuentadealtocosto.org/), (2) Observatorio de Diabetes de Colombia (http://www.odc.org.co/), and (3) the Sistema Integral de Información de la Protección Social databases (https://www.minsalud.gov.co/salud/Paginas/SistemaIntegraldeInformaci%C3%B3nSISPRO.aspx) (Fig. 4). Data on SES indicators was collected from the Departamento Administrativo Nacional de Estadística (DANE)^37^ and Instituto Colombiano de Bienestar Familiar^38^ (Table 2).

**Table 2:** Comparison of socio-economic status (SES) indicators for Chocó and Antioquia.

Measure^1^	Chocó	Antioquia
Human Development Index (HDI)^2^	0.73	0.85
Literacy Rate	76%	89%
GDP (per capita)^3^	$6 M	$16 M
Life Expectancy	68 yrs	73 yrs
Employment Rate	77%	88%
Modern Housing Rate	10%	79%
Protein Consumption Deficit	57%	26%
Calcium Deficit	95%	75%

^1^SES index data taken from the Colombian census^37^ and the Colombian national nutritional survey^38^.

^2^The HDI is a composite of measure of health, education and standard of living.

^3^Gross domestic product (GDP) estimates are shown as millions of Colombian pesos (COP).

### Data availability

Genome sequence variant data are available from the project resources listed in Table 1. Genotype data for Chocó are available by request under the terms of a data use agreement managed by UTCH.

## Results

### Comparative genetic ancestry

Here and elsewhere^19,22^, we characterized the genetic heritage of Chocó and Antioquia with respect to their populations’ ancestry proportions derived from Africa, Europe and the Americas. To do so, whole genome genotypes characterized for donors from Chocó, along with publicly available whole genome sequences from Antioquia, were compared to genomes from putative ancestral source populations collected from a variety of sources (Table 1). Details of the approaches we used for all comparative genomic analyses can be found in the Methods section. Pairwise genomic distances projected onto two dimensions group individuals from Chocó with an African population from Nigeria, whereas individuals from Antioquia group most closely with a European population from Spain (Fig. 1A). Nevertheless, both populations show visual evidence of substantial admixture among the three major continental population groups on this same plot. The inferred continental genetic ancestry fractions for Chocó and Antioquia are also largely consistent with the states’ demographic profiles, which were gleaned from self-reported ethnicity, with Chocó having predominantly African ancestry and Antioquia having mainly European ancestry. Admixture analysis revealed that the population of Chocó has 76% African, 13% European, and 11% Native American ancestry, whereas Antioquia has 75% European, 18% Native American and 7% African ancestry (Fig. 1B and Supplementary Figure 2).

### Comparative T2D genetic risk

We asked whether the differences in genetic ancestry between Chocó and Antioquia are related to population-specific genetic risk for diabetes by comparing the distributions of known T2D risk alleles for the two populations using the previously described genomic datasets. T2D risk alleles for a total of 165 single nucleotide polymorphisms (SNPs) were mined from a collection of 29 T2D genome-wide association studies (GWAS) (Supplementary Table 1). Population-specific frequencies of the risk and non-risk alleles for each T2D-associated SNP were measured and used to calculate a log odds ratio (*OR*) that expresses the relative genetic risk of T2D for the two populations: Chocó/Antioquia. Log odds ratios were used to provide a statistical framework to measure the T2D risk contributions of individual SNPs and to allow for a meta-analysis that considers the additive genetic risk contribution of all SNPs together. Details of this approach are provided in the Methods section. The majority of T2D associated SNPs show higher risk allele frequencies in Chocó compared to Antioquia, pointing to a relatively higher genetic risk of T2D in the population of Chocó (Fig. 2A). Ninety one (91) individual SNPs show significant differences in risk versus non risk allele frequencies in Chocó compared to Antioquia; 62 (68%) of those SNPs reflect significantly greater T2D genetic risk in Chocó compared to only 29 (32%) with higher risk in Antioquia. When all of the T2D-associated SNPs are considered together using meta-analysis, Chocó shows significantly greater population-wide genetic risk for T2D than Antioquia. Chocó/Antioquia T2D meta-analysis *OR* values, along with their 95% confidence intervals, were computed using both fixed and random effect models as well as via bootstrap analysis. All three approaches show significantly higher T2D genetic risk in Chocó compared to Antioquia (Fig. 2A).

We performed a series of controls in an effort to ensure that the difference observed for T2D genetic risk between Chocó and Antioquia cannot be attributed to any systematic bias in the SNP allele frequencies of the two populations (Methods). First, we used bootstrap analysis of the T2D SNP set to evaluate the signal-to-noise ratio in the data. In particular, we wanted to assess whether the observed difference in T2D genetic risk between Chocó and Antioquia may be due to a few outlier SNPs (Supplementary Figure 4). Sampling with replacement from the set of T2D-associated SNPs was used to generate 10,000 replicate T2D SNP sets, each of which was used to calculate a meta-analysis *OR* value. The distribution of bootstrap replicate *OR* values is centered around observed *OR* value, and the mean bootstrap *OR* value is significantly greater than 0 (Fig. 2B; z = −3.99, *P* = 6.6 × 10^−5^). The results of the bootstrap analysis are consistent with greater T2D genetic risk in Chocó and indicate that the signal in the data, based on the individual SNP *OR* values, is robust to sampling noise.

We next addressed whether the observed difference in T2D genetic risk can be attributed to a systematic bias in the allele frequencies for disease-associated SNPs between the two populations. This is particularly relevant given the fact that the vast majority of GWAS are conducted on populations of European ancestry, more similar to what is seen for Antioquia. In fact, it has recently been shown that attempts to compare genetic risk between populations with divergent ancestry profiles can be confounded by demographic factors that yield differences in the overall frequencies of risk alleles; effects of this kind can in turn lead to systematic biases in population-specific genetic risk estimates^39^. We attempted to control against this possibility using the two approaches described below.

We developed a simulation-based approach in order to control for the possible effects of demographic history on estimates of population-specific T2D genetic risk for Chocó and Antioquia. If the apparent elevated genetic risk for T2D in Chocó reflects a bias in the relative frequencies of disease-associated SNPs, perhaps owing to increased African ancestry of the population, then we would expect to see an overall shift to higher estimated disease risk for Chocó compared to Antioquia. To evaluate this possibility, 500,000 SNP sets of the same size as the set of T2D-associated SNPs were randomly simulated from a collection of disease-associated SNPs taken from the NHGRI-EBI GWAS catalog^30^. For each of these random SNP sets, a meta-analysis of the SNP relative genetic risk log odds ratios (Chocó/Antioquia) was performed, yielding a random meta-analysis log odds ratio value (*OR*). The null distribution of the resulting random meta-analysis *OR* values was then compared to the observed T2D relative genetic risk *OR* value for Chocó/Antioquia. Contrary to the expectations of the demographic bias model, Antioquia shows a higher overall relative genetic risk when ensembles of randomly sampled disease-associated SNP sets are analyzed (Fig. 2C). In addition, the observed T2D relative genetic risk *OR* value for Chocó/Antioquia is significantly greater than the expected *OR* value based on the null distribution, further validating the observed elevated genetic risk for T2D in Chocó (*z* = 4.0, *P* = 6.3 × 10^−5^).

In addition to the simulation-based approach described above, we also used disease-associated SNPs from the NHGRI-EBI GWAS catalog to compute the relative genetic risk between Chocó and Antioquia for 324 additional diseases. In this case, a systematic bias in the population-specific allele frequencies of disease-associated SNPs would be expected to reveal an overall elevation of disease genetic risk in one of the two populations. However, the distribution of the differences in predicted genetic risk for these diseases is centered very close to 0 and more or less symmetrical (Fig. 2D); the mean genetic risk difference (Chocó - Antioquia) for these diseases is not significantly different than 0 (*z* = −0.1, *P* = 0.92). Taken together, these three controls suggest that the observed difference in T2D genetic risk for Chocó versus Antioquia cannot be attributed to any systematic bias in disease-associated allele frequencies between the two populations.

### Genetic ancestry and T2D risk

Considering their respective ancestry profiles, the higher T2D genetic risk that we observe for the population of Chocó compared to Antioquia is consistent with previous results showing a correlation between African genetic ancestry and T2D prevalence in the US^12^. We asked whether elevated genetic risk of T2D in Chocó may also be related to greater African ancestry, and conversely lower European ancestry, in Chocó compared to Antioquia. To do this, we computed polygenic T2D risk scores for individuals from Chocó and Antioquia along with individuals from their most closely related putative ancestral populations in Europe (Spain) and Africa (Nigeria). We applied a widely used approach that computes polygenic risk scores for individual genomes, or whole genome genotypes, based on the sum of risk alleles present across all associated SNPs^40–43^ (Methods). The Antioquia population has the lowest T2D genetic risk measured this way followed by the Spanish population; however, the T2D genetic risk score distributions between these two populations are not significantly different (*t* = 0.3, *P* = 0.8; Fig. 3A). Chocó has significantly greater T2D genetic risk than Antioquia (*t* = 5.7, *P* = 4.1 × 10^−8^), and the Nigerian population has the highest overall risk (Fig. 3A). Thus, the T2D genetic risk score distributions for these populations follow the increasing proportions of African ancestry, and decreasing European ancestry, seen among them. We also performed a similar analysis of T2D genetic risk analyses for a pair of African-American and European-American populations from the US, and find the same patterns of elevated T2D genetic risk associated with African ancestry that we see for Colombia, consistent with previous results^12^ (Supplementary Figure 5). Finally, we show that the African ancestry percentages for individuals from Colombia and the US are positively correlated with their polygenic risk scores for T2D (*r* = 0.81, *P* = 2.7 × 10^−44^; Fig. 3B).

We further evaluated the relationship between genetic ancestry and T2D genetic risk worldwide by comparing five European populations to seven African populations (Fig. 3C). All of the African populations have higher T2D genetic risk than the European populations, and the difference between the African versus European ancestry group T2D genetic risk averages is highly significant (*t* = 33.9, *P* = 1.4 × 10^−164^). These results lend additional support to the association of African genetic ancestry with elevated T2D genetic risk.

### Observed T2D prevalence

Given the elevated genetic risk for T2D in the Afro-Colombian population of Chocó, along with its association with African ancestry, we expected to see a substantially higher prevalence of diabetes in Chocó compared to Antioquia. Indeed, numerous studies report that African-Americans in the US have far higher prevalence of T2D than European-Americans^8–10^. However, we were surprised to find that the reported prevalence of diabetes is in fact more than three-times higher in Antioquia than in Chocó (Fig. 4A). Averaging data from three separate epidemiological database sources, maintained by governmental and non-governmental organizations, shows Antioquia with an age-adjusted diabetes prevalence of 1.9%, which is the 4^th^ highest out of 32 states in the country, compared to 0.6% for Chocó, which is ranked 27^th^. The large difference in diabetes prevalence observed for Chocó versus Antioquia is highly consistent across the three different Colombian epidemiological databases that we sourced (Fig. 4B).

The far lower prevalence of diabetes in Chocó versus Antioquia, compared to what may be expected based on the genetic profiles of their populations, strongly suggests that environmental factors predominantly shape diabetes outcomes in the region. This would be consistent with several large cohort studies showing that environmental factors contribute substantially more to T2D than genetic factors^44–46^, and the populations of Chocó and Antioquia do indeed occupy very distinct environments. In particular, as previously stated, the population of Chocó has far lower overall SES compared to Antioquia (Table 2). For example, the per capita gross domestic product in Chocó is almost three times lower than that of Antioquia. Chocó also has lower levels of literacy, life expectancy, employment, and modern housing along with higher dietary deficits of protein and calcium than Antioquia. Considered together, these factors give Chocó a human development index (HDI) of 0.73, ranked 31^st^ out of 32 Colombian states, compared to an HDI of 0.85 for Antioquia, which ranks 4^th^ in the country. Thus, it appears that even though low SES has been associated with the risk for T2D in numerous studies^47^, in Chocó low SES somehow serves as a protective factor against T2D. This unexpected finding suggests that poverty may play a very different role in the etiology of complex disease, particularly for diabetes and perhaps other metabolic syndrome disorders, in Colombia compared to more developed countries in the Global North.

## Discussion

Our study of the contributions of genetic ancestry and environmental factors to T2D prevalence in two divergent Colombian populations suggests that poverty can serve as a T2D protective factor in Colombia. The possibility that poverty in Chocó is an environmental protective factor against T2D, as opposed to a strong risk factor as seen for African-Americans in the US, may be attributed to the differing nature of poverty in developed countries compared to some parts of the developing world. Poverty in the US is associated with poor diet and other lifestyle factors that elevate T2D prevalence^16,17,48,49^. However, poverty in Chocó, which is generally more extreme than what is found in the US, is actually associated with a diet that is protective against T2D, particularly when compared to Antioquia. The dietary staples of Chocó are fish, plantains, yucca and rice; fish are readily available from the Atrato River and its tributaries, and plantains and yucca are cultivated along the banks of this vast river system^50,51^. Thus, the typical diet of Chocó is high in polyunsaturated lipids, such as omega-3 and omega-6 fatty acids, and fiber, both of which are known to mitigate T2D risk. In Antioquia, the main sources of protein are beef and pork, which are rich in both cholesterol and triglycerides formed by saturated fatty acids, known risk factors for T2D. In addition to the ready availability of fish in the region, SES in Chocó also impacts dietary choices in a way that is protective against T2D. In Quibdó, the capital of Chocó, one kilogram of meat costs $9000 Colombian pesos, or approximately $3 US dollars; 10 kg of fish from the Atrato River can be bought for the same amount, providing a week’s worth of protein.

We also found Chocó and Antioquia to be distinct with respect to the prevalence of alcohol consumption and tobacco use, both of which have been implicated as environmental factors that influence T2D outcomes. A 2013 government survey on the consumption of psychoactive substances in Colombia found that Chocó had the highest prevalence of alcohol consumption for the country, with 44.6% of respondents reporting alcohol consumption over the past 30 days compared to 36.6% for Antioquia^52^. Since moderate alcohol consumption has been linked to reduced risk for the onset of T2D^53–55^, this could represent an additional protective factor associated with the lifestyle in Chocó. Conversely, Antioquia was found to have higher tobacco use in the same survey, with 14.1% use over the last month compared to 6.6% for Chocó. Smoking is a known risk factor for T2D^56–58^, pointing to yet another possible advantage of the lifestyle in Chocó with respect to T2D prevalence.

Another way to consider the discordant results that we observed for population-specific genetic risk versus the observed prevalence of diabetes in Colombia is through the lens of economic development as opposed to poverty *per se*. While the notion that poverty in Chocó serves as a protective factor against diabetes was certainly unexpected to us, if we consider Chocó to be under-developed relative to Antioquia, then the environmental protective effect may not be as surprising. Indeed, as previously stated, the HDI for Chocó points to substantial under-development compared to the rest of the country, and the pyramid shaped age distribution of Chocó is more consistent with what is seen in less developed countries; the narrower age distribution of Antioquia, on the other hand, resembles those of more developed countries (Supplementary Figure 6). T2D has been considered to be a disease of the developed world, as it is generally more prevalent in industrialized than less-developed countries^59^. In fact, studies have shown precipitous increases in T2D prevalence in populations that have undergone rapid transitions to more developed economies^60^. The comparison of Chocó versus Antioquia may underscore the public health relevance of stark differences in economic development within a single country, albeit in way that counterintuitively favors the less developed region.

It is also worth noting that Chocó is more rural, and less urbanized, than Antioquia. Chocó is relatively underpopulated with a population density of 11 individuals per km^2^ compared to 99 per km^2^ for Antioquia. The rural setting of Chocó, along with the overall challenging conditions of its environment, are associated with a more physically active lifestyle compared to more modernized parts of the country^50,51^, highlighting yet another potentially protective factor against T2D. Interestingly, a recent study in India showed that low SES is simultaneously a risk factor for T2D in cities and a protective factor for T2D in more rural areas^61^. Thus, it may be the case that urban poverty in developing countries is more reminiscent of overall poverty in the developed world, in terms of risk for T2D, whereas the features of rural poverty in the developing world are distinctive and protective for T2D.

We explored the relationship between economic development and T2D for the entire country by comparing HDI levels to T2D prevalence estimates for all states. We observe a strong positive correlation between HDI and T2D prevalence across Colombia, with more developed regions of the country showing higher T2D prevalence estimates (Supplementary Figure 7A). This finding is consistent with the notion that lower levels of development within the country can serve as a protective factor against T2D. However, it could also be taken to suggest the possibility that the lower prevalence of T2D in Chocó reflects a bias in disease reporting, owing to lower SES and accordingly reduced access to healthcare services. We evaluated this possibility by comparing prevalence estimates for 43 diseases between Chocó and Antioquia. A reporting bias for Chocó, based on reduced access to healthcare, would be expected to reveal itself as an overall reduction in prevalence estimates for numerous diseases. In fact, Chocó shows greater prevalence for 24 diseases, compared to 19 for Antioquia, and the difference between the two is not statistically significant (Supplementary Figure 7B). These results indicate that a reporting bias based on differential access to healthcare does not likely explain the lower prevalence of T2D observed for Chocó.

Genomic approaches to health care, while still in their infancy in the region, hold great promise for Latin America, especially as the public health burden continues to shift toward common complex diseases with at least partial genetic etiology. The distinction that we observe between population-specific genetic risk and observed prevalence of T2D provides lessons for the implementation of genomic approaches to personalized and precision medicine in Latin American countries such as Colombia. Caution should be taken when extrapolating results from studies in the Global North, where the vast majority of this kind of research is still conducted^62–64^, to developing countries in Latin America. For instance, a commonly accepted environmental risk factor for many common diseases, such as SES, may have very different implications in Latin America compared to the US. In addition, the public health value of dietary and lifestyle choices, which may have been historically dictated by poverty, should be recognized and incorporated into public health campaigns as countries in Latin America continue to experience rapid economic development and urbanization. A corollary of this suggestion would be to strategically avoid pitfalls of urbanization in the developed world, such as the increasingly sedentary lifestyle, the reliance on processed and fast foods as well as the emergence of so-called ‘food deserts’ in poor neighborhoods where it is exceedingly difficult, if not impossible, to access fresh and whole foods.

Finally, caution also needs to be exercised when extrapolating the results of studies on the genetic architecture of complex diseases between populations with distinct ancestry profiles^39^. Genetic associations discovered in one population may not replicate in a different population, and ancestry and admixture can have additional confounding effects on the expression of genetic variants. Nevertheless, as we have endeavored to show here, exploration of disease associated variants in understudied populations can provide valuable insight into the joint contributions of genetics and environment to common complex diseases, which are an increasing public health threat to the developing economies of the Global South.

## Electronic supplementary material

Supplementary Information
