# Supplementary material for: Influence of genetic ancestry and socioeconomic status on type 2 diabetes in the diverse Colombian populations of Chocó and Antioquia
Source: Sci Rep. 2017 Dec 7;7:17127. doi: 10.1038/s41598-017-17380-4 (PMC5719455; doi:10.1038/s41598-017-17380-4)
Supplement: Supplementary file 1 — Supplementary Information [file 41598_2017_17380_MOESM1_ESM.pdf]

Supplementary Information For:

Influence of genetic ancestry and socioeconomic status on type 2 diabetes in the diverse Colombian populations of Chocó and Antioquia

Aroon T. Chande[1,2,3], Jessica Rowell[1], Lavanya Rishishwar[1,2,3], Andrew B. Conley[2], Emily T. Norris[1,2,3], Augusto Valderrama-Aguirre[3,4], Miguel A. Medina-Rivas[3,5] and I. King Jordan[1,2,3,*]

[1] School of Biological Sciences, Georgia Institute of Technology, Atlanta, Georgia, USA

[2] IHRC-Georgia Tech Applied Bioinformatics Laboratory, Atlanta, Georgia, USA

[3] PanAmerican Bioinformatics Institute, Cali, Valle del Cauca, Colombia

[4] Biomedical Research Institute, Universidad Libre, Cali, Valle del Cauca, Colombia

[5] Centro de Investigación en Biodiversidad y Hábitat, Universidad Tecnológica del Chocó, Quibdó, Chocó, Colombia

*Corresponding author:
I. King Jordan
School of Biological Sciences
Georgia Institute of Technology
950 Atlantic Drive
Atlanta, Georgia 30332
USA
404-385-2224
king.jordan@biology.gatech.edu

Supplementary Figures and Table

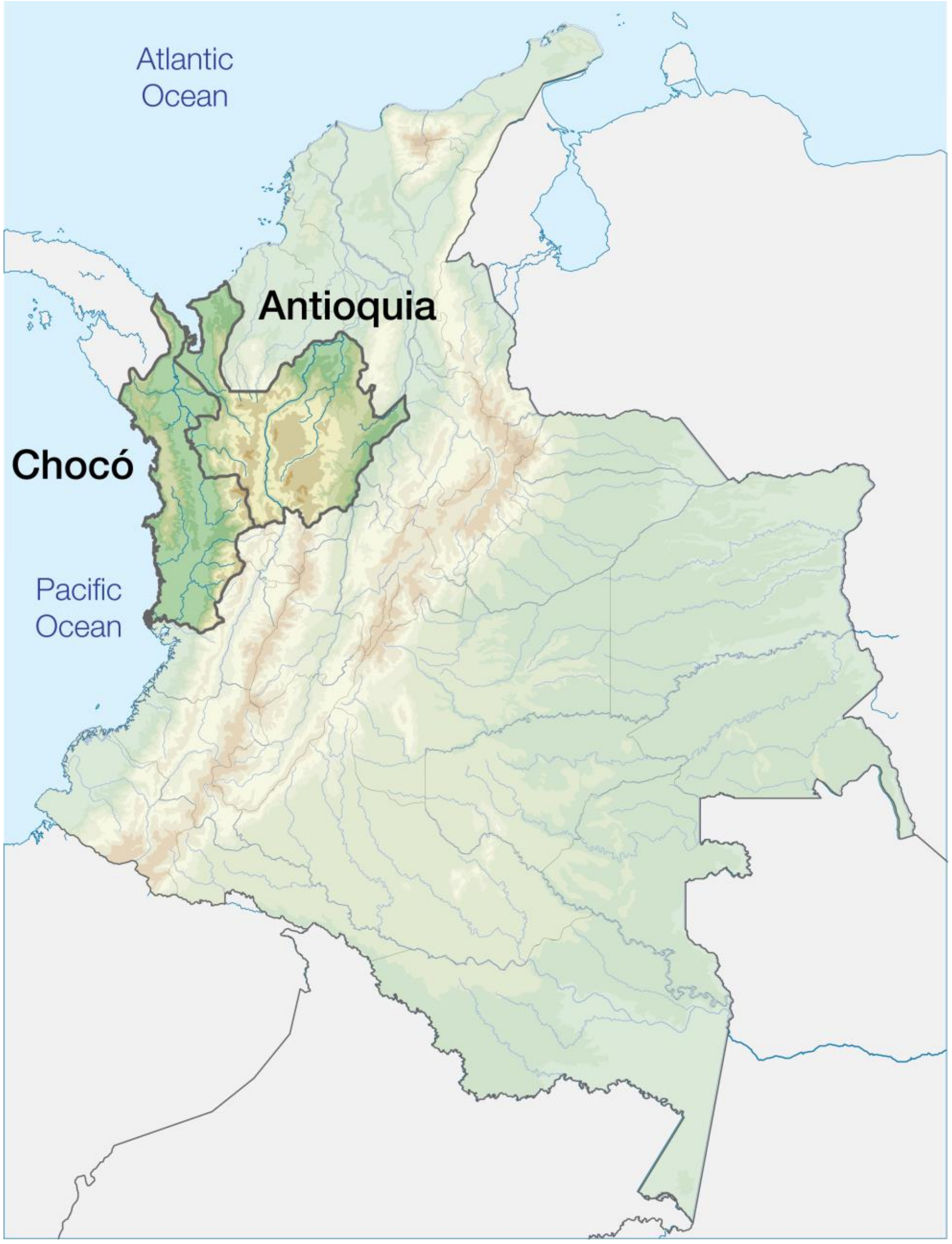

Supplementary Figure 1. **Relief map of Colombia showing the locations of the administrative departments (*i.e.*, states) of Chocó and Antioquia.** Map adapted from https://commons.wikimedia.org/wiki/File:Mapa_de_Colombia_(relieve).svg, edited to highlight the states of interest. The image file is licensed under the Creative Commons Attribution-Share Alike 3.0 Unported license https://creativecommons.org/licenses/by-sa/3.0/deed.en.

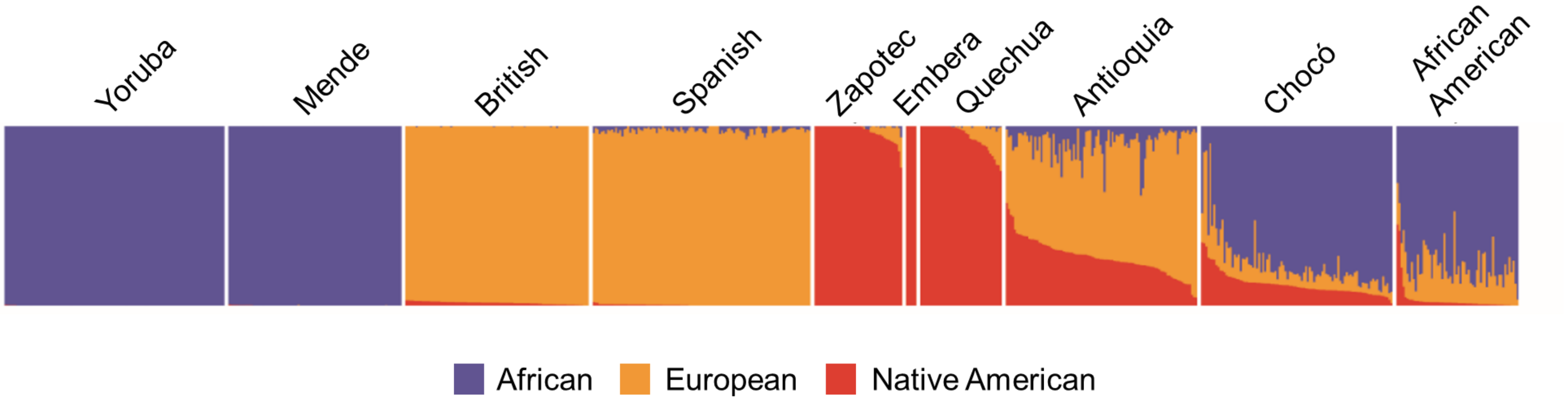

Supplementary Figure 2. **Admixture bar chart showing the percentage of African (blue), European (orange) and Native American (red) ancestry for the individuals from Antioquia Chocó analyzed here.** Admixture was run with K=3 clusters, corresponding to the three continental ancestry groups, using the global putative ancestral source populations shown here[2].

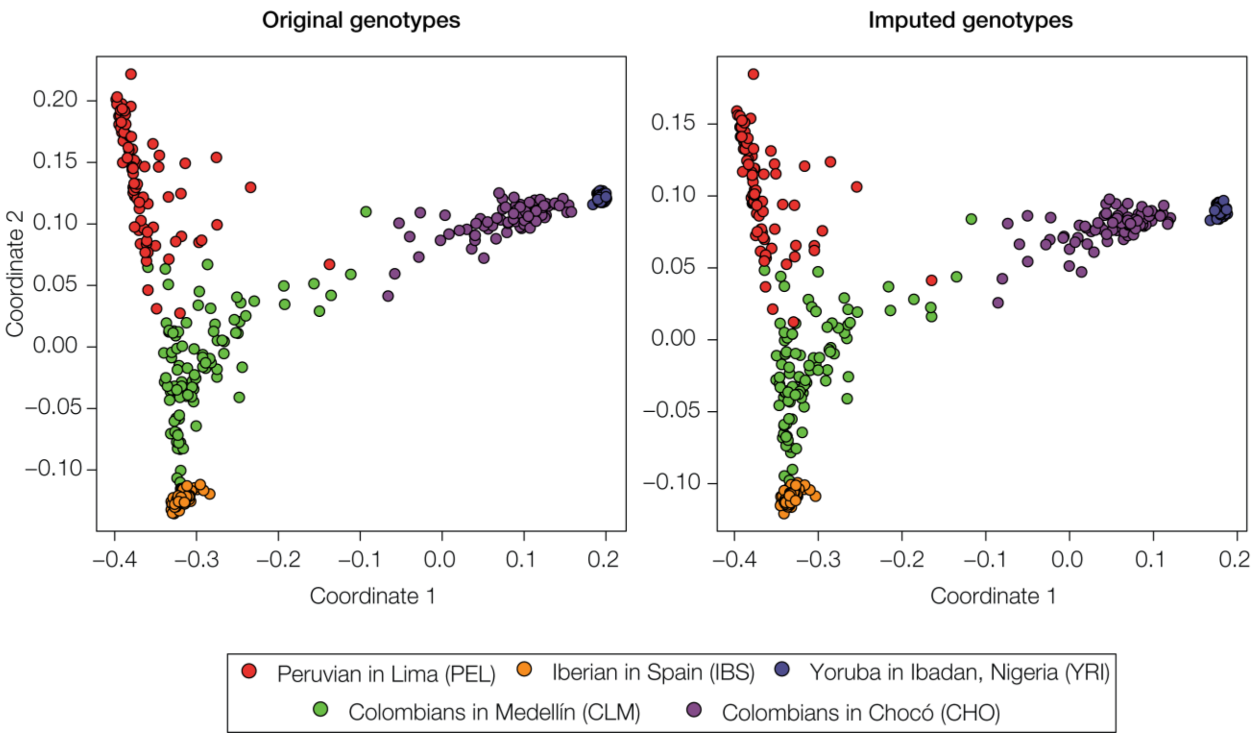

Supplementary Figure 3. **Validation of the SNP imputation process for the Chocó genotypes via comparison of genetic ancestry patterns before (original genotypes) and after (imputed genotypes) imputation.** Pairwise genomic distances between individuals from Chocó and a panel of global reference populations (see color key) characterized via whole genome sequencing as part of the 1KGP are shown before and after imputation.

Supplementary Table 1. **Type 2 diabetes (T2D) associated SNPs analyzed in this study.** 165 T2D-associated SNPs, corresponding to 29 studies, were taken from the NHRGI-EBI GWAS database[1].

rsID[1]	Chr[2]	Pos[2]	Risk Allele[3]	Gene(s)[4]	PubMed ID[5]
rs5945326	X	153634467	A	DUSP9	20581827
rs17106184	1	50444313	G	FAF1	24509480
rs11165354	1	91728765	A	TGFBR3	23209189
rs7542900	1	94604485	C	SLC44A3, F3	22238593
rs10923931	1	119975336	T	NOTCH2, ADAM30	18372903
rs2075423	1	213981376	G	PROX1	24509480
rs2820446	1	219575476	C	LYPLAL1	24509480
rs6426514	1	228744368	A	RHOU	23300278
rs12027542	1	233204408	A	PCNXL2	21490949
rs679992	1	241024982	T	intergenic	25102180
rs10190052	2	646674	C	TMEM18	24509480
rs11677370	2	3793830	T	intergenic	21490949
rs12613372	2	30845153	G	GALNT14, CAPN13	25102180
rs7578597	2	43505684	T	THADA	18372903
rs243088	2	60341610	T	BCL11A	24509480
rs243021	2	60357684	A	BCL11A	20581827
rs73954691	2	127663671	G	LIMS2	25483131
rs6723108	2	134722410	T	TMEM163	23209189
rs7560163	2	150781422	C	RBM43, RND3	22238593
rs3923113	2	164645339	A	GRB14	21874001
rs2943640	2	226228869	C	IRS1	24509480
rs17036101	3	12236345	G	SYN2, PPARG	18372903
rs13081389	3	12248301	A	PPARG	20581827
rs1801282	3	12351626	C	PPARG	17463246
rs6780569	3	23156993	G	UBE2E2	23945395
rs7612463	3	23294959	C	UBE2E2	24509480
rs831571	3	64062621	c	PSMD6	22158537
rs4607103	3	64726228	C	ADAMTS9	18372903
rs2063640	3	102484201	A	ZPLD1	21490949
rs11708067	3	123346931	A	ADCY5	22693455
rs11717195	3	123363551	T	ADCY5	24509480
rs3773506	3	142712158	C	PLS1	21490949
rs7630877	3	179943530	A	PEX5L	21490949
rs4402960	3	185793899	T	IGF2BP2	17463246
rs1470579	3	185811292	C	IGF2BP2	20581827
rs6769511	3	185812502	C	IGF2BP2	18711366
rs1374910	3	185813873	T	IGF2BP2	21573907
rs16861329	3	186948673	C	ST64GAL1	24509480

rs6808574	3	188022735	C	LPP	24509480
rs6815464	4	1316113	C	MAEA	22158537
rs4458523	4	6288259	G	WFS1	24509480
rs1801214	4	6301295	T	WFS1	20581827
rs7659604	4	121744359	T	NR	17554300
rs6813195	4	152599323	C	TMEM154	24509480
rs702634	5	53975590	A	ARL15	24509480
rs10461617	5	56808481	A	MAP3K1	23209189
rs4457053	5	77129124	G	ZBED3	20581827
rs319598	5	134904545	C	PCBD2	24509480
rs17053082	5	155967220	T	intergenic	23300278
rs9295474	6	20652486	G	CDKAL1	21490949
rs4712523	6	20657333	G	CDKAL1	19401414
rs4712524	6	20657634	G	CDKAL1	18711366
rs10946398	6	20660803	C	CDKAL1	17463249
rs7754840	6	20661019	C	CDKAL1	17463246
rs7756992	6	20679478	G	CDKAL1	17460697
rs10440833	6	20687890	A	CDKAL1	20581827
rs6931514	6	20703721	G	CDKAL1	18372903
rs9465871	6	20717024	C	CDKAL1	17554300
rs2244020	6	31379674	G	HLA-B	25102180
rs3916765	6	32717773	A	HLA-DQA2	22693455
rs9470794	6	38139068	C	ZFAND3	22158537
rs1535500	6	39316274	T	KCNK16	22158537
rs9472138	6	43844025	T	VEGFA	18372903
rs1048886	6	70579486	G	C6orf57	21490949
rs4273712	6	126643364	G	C6orf173	24509480
rs6937795	6	136970143	A	IL20RA	24509480
rs642858	6	139952510	A	intergenic	21490949
rs7795991	7	13861106	G	ETV1	24509480
rs17168486	7	14858657	T	DGKB	24509480
rs864745	7	28140937	T	JAZF1	18372903
rs849134	7	28156603	A	JAZF1	20581827
rs849135	7	28156794	G	JAZF1	24509480
rs10231619	7	43280995	T	HECW1	25102180
rs7636	7	100892456	A	ACHE	21490949
rs6467136	7	127524904	G	PAX4, GCC1	22158537
rs10229583	7	127606849	G	ARF5, PAX4, SND1	23532257
rs791595	7	128222749	A	MIR129, LEP	23945395
rs972283	7	130782095	G	KLF14	20581827
rs516946	8	41661730	C	ANK1	24509480

rs7003257	8	67701155	T	CPA6	25102180
rs17359493	8	94844683	G	INTS8	25102180
rs7845219	8	94925274	T	TP53INP1	24509480
rs896854	8	94948283	T	TP53INP1	20581827
rs13266634	8	117172544	C	SLC30A8	17293876
rs3802177	8	117172786	G	SLC30A8	20581827
rs1561927	8	128555832	C	TMEM75	24509480
rs4527850	8	133184606	T	WISP1	23300278
rs5219	9	22029548	T	KCNJ11	17463246
rs2383208	9	22132077	A	CDKN2A, CDKN2B	19401414
rs11257655	10	12265895	T	CDC123	22961080
rs10906115	10	12272998	A	CDC123, CAMK1D	20862305
rs12779790	10	12286011	G	CDC123, CAMK1D	18372903
rs2812533	10	69692529	C	C10orf35	24509480
rs12571751	10	79182874	A	ZMIZ1	24509480
rs10788575	10	88008827	A	PTEN	24509480
rs6583826	10	92588073	G	KIF11	21490949
rs1111875	10	92703125	C	HHEX	17463246
rs5015480	10	92705802	C	HHEX	17463249
rs34872471	10	112994312	C	TCF7L2	25483131
rs7901695	10	112994329	C	TCF7L2	17463249
rs4506565	10	112996282	T	TCF7L2	17554300
rs10886471	10	119389891	C	GRK5	22961080
rs10510110	10	122432914	C	PLEKHA1	24509480
rs10741243	10	131149699	G	TCERG1L	21490949
rs3842770	11	2157440	A	INS-IGF2	25102180
rs11043007	11	2183058	G	ASCL2, TH	25102180
rs231362	11	2670241	G	KCNQ1	20581827
rs231356	11	2684113	T	KCNQ1	25102180
rs2237892	11	2818521	C	KCNQ1	18711367
rs163182	11	2822986	C	KCNQ1	21799836
rs163184	11	2825839	G	KCNQ1	24509480
rs2283228	11	2828300	A	KCNQ1	25102180
rs2237895	11	2835964	C	KCNQ1	20174558
rs2237897	11	2837316	C	KCNQ1	18711366
rs2722769	11	11206827	C	GALNTL4, LOC729013	22238593
rs5215	11	17387083	C	KCNJ11	17463249
rs9300039	11	41893816	C	intergenic	17463248
rs1552224	11	72722053	A	CENTD2	20581827
rs1387153	11	92940662	T	MTNR1B	20581827
rs10830963	11	92975544	G	MTNR1B	24509480

rs7107217	11	129603795	C	TMEM45B, BARX2	22238593
rs10842994	12	27812217	C	KLHDC5	24509480
rs12304921	12	50963759	G	NR	17554300
rs1153188	12	54705212	A	DCD	18372903
rs1531343	12	65781114	C	HMGA2	20581827
rs2261181	12	65818538	T	HMGA2	24509480
rs343092	12	65857160	T	HMGA2	25102180
rs1495377	12	71183321	G	NR	17554300
rs4760790	12	71241014	A	LGR5, TSPAN8	20581827
rs7961581	12	71269322	C	LGR5, TSPAN8	18372903
rs7305618	12	120965129	C	HNF1A	21573907
rs12427353	12	120989098	G	HNF1A	24509480
rs7957197	12	121022883	T	HNF1A	20581827
rs1727313	12	123156306	C	MPHOSPH9	24509480
rs10507349	13	26207391	G	RNF6	24509480
rs1359790	13	80143021	G	SPRY2	20862305
rs730570	14	100676553	G	C14orf70	21573907
rs7403531	15	38530704	T	RASGRP1	22961080
rs335810	15	60076007	A	ANXA2	25102180
rs7163757	15	62099409	C	C2CD4A	24509480
rs7172432	15	62104190	A	C2CD4A	20818381
rs1436955	15	62112183	C	C2CD4B	20862305
rs7178572	15	77454848	G	HMG20A	22693455
rs7119	15	77485290	T	HMG20A	21490949
rs11634397	15	80139880	G	ZFAND6	20581827
rs2028299	15	89831025	C	AP3S2	21874001
rs8042680	15	90978107	A	PRC1	20581827
rs12899811	15	91000846	G	PRC1	24509480
rs8050136	16	53782363	A	FTO	17463248
rs9936385	16	53785257	C	FTO	24509480
rs9939609	16	53786615	A	FTO	17554300
rs11642841	16	53811575	A	FTO	20581827
rs17797882	16	79373021	T	WWOX	22158537
rs623323	17	796780	T	NXN	23300278
rs312457	17	7037074	G	SLC16A13	23945395
rs4430796	17	37738049	G	HNF1B, TCF2	20581827
rs10460009	18	2948031	C	LPIN2	21490949
rs8090011	18	7068463	G	LAMA1	22693455
rs275856	18	24259188	T	OSBPL1A	25483131
rs12970134	18	60217517	A	MC4R	24509480
rs3786897	19	33402102	A	PEPD	22158537

rs6017317	20	44318326	G	FITM2, R3HDML, HNF4A	22158537
rs4812829	20	44360627	A	HNF4A	21874001
rs328506	20	57454548	C	CTCFL, RBM38, HMG1L1, PCK1	23300278
rs2833610	21	32012873	A	HUNK	21490949

[1]T2D-associated SNP identifier numbers, from the NCBI Single Nucleotide Polymorphism database (dbSNP https://www.ncbi.nlm.nih.gov/projects/SNP/).

[2]Chromosomal locations for the T2D-associated SNPS, corresponding to the GRh37/h19 version of the human genome reference sequence.

[3]Identity of the risk alleles (nucleotide variants) that are associated with higher risk of T2D for each SNP. Risk allele identities are shown here for the positive DNA strand.

[4]HGNC gene symbols for the genes considered to be influenced by the T2D-associated SNPs, according to each study.

[5]NCBI PubMed identifier numbers for the publications where each T2D-associated SNP was reported.

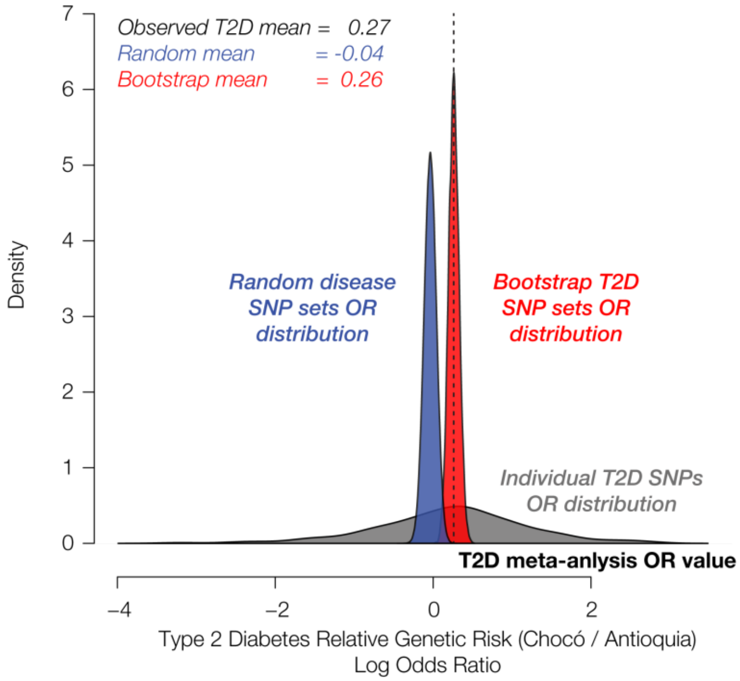

Supplementary Figure 4. **Distributions of T2D SNP OR values along with control analysis distributions.** The distribution of OR values for the 165 individual T2D-associated SNPs is shown in gray, and the observed T2D meta-analysis OR value is indicated with a dashed line. The bootstrap T2D SNP set OR value distribution is shown in red, and the random disease-associated SNP set OR value distribution is shown in blue. Mean values for all three distributions are shown.

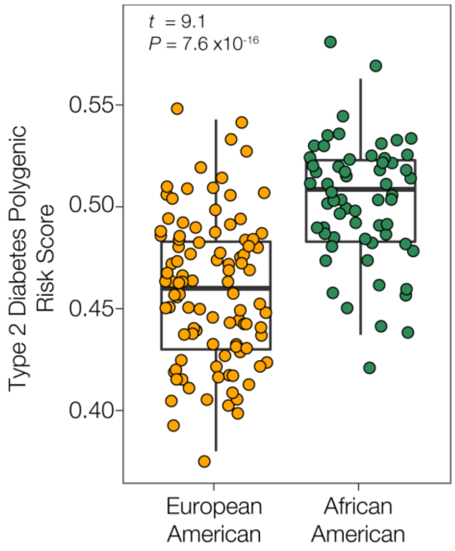

Supplementary Figure 5. **Type 2 diabetes polygenic risk score distributions for European-American (orange) and African-American (green) populations from the US[3].** The significance of the difference between the two distributions, based on the Student's t-test, is shown.

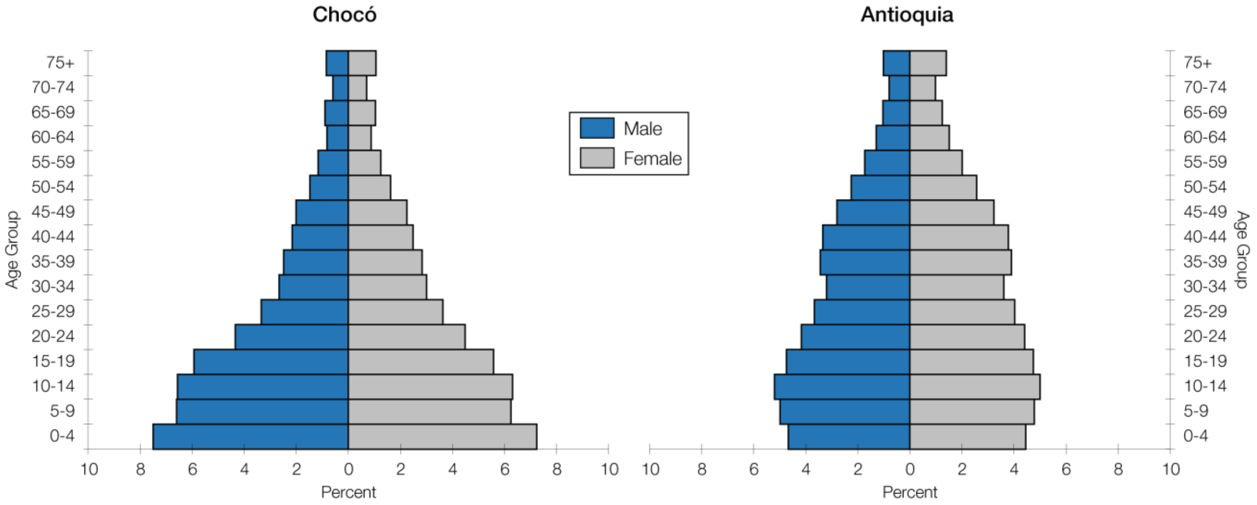

Supplementary Figure 6. **Age pyramids for Chocó and Antioquia.** The percentages of males and females in each population are shown for different age groups[4].

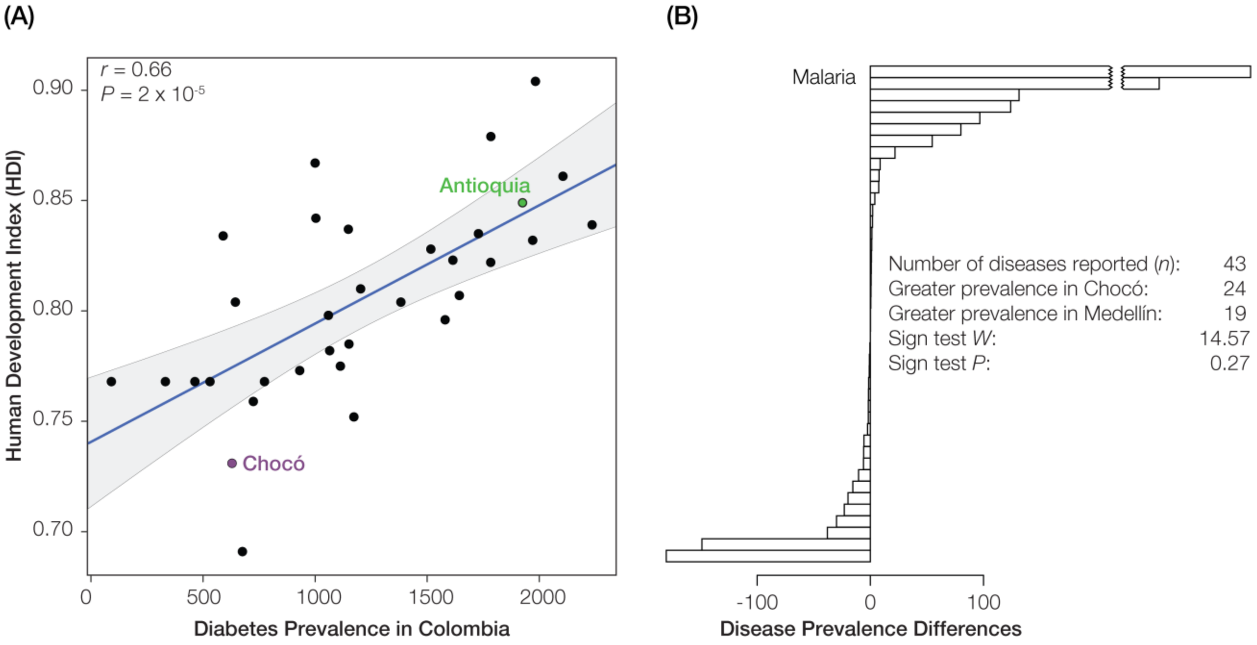

Supplementary Figure 7. **Economic development and disease prevalence reporting in Chocó and Antioquia.** (A) Regression of the human development index (HDI, y-axis) against diabetes prevalence estimates (x-axis) for Colombian administrative departments (*i.e.,* states). The linear trend line is shown in blue with 95% CI in gray. The values of r and P for the Pearson correlation coefficient of the regression are shown. (B) Disease prevalence estimate differences (Chocó – Antioquia) for 43 reported diseases. The values of W and P for a binomial sign test of consistent differences between pairs of disease prevalence estimates are shown. Note that the x-axis breaks at the two highest values for malaria, which has far higher prevalence in Chocó.

Supplementary References

1. Welter D*, et al.* The NHGRI GWAS Catalog, a curated resource of SNP-trait associations. *Nucleic Acids Res* **42**, D1001-1006 (2014).

2. Medina-Rivas MA*, et al.* Choco, Colombia: a hotspot of human biodiversity. *Rev Biodivers Neotrop* **6**, 45-54 (2016).

3. Genomes Project C*, et al.* A global reference for human genetic variation. *Nature* **526**, 68-74 (2015).

4. Uribe Vélez A, Maldonado Gómez H, Fernández Ayala PJ, Vargas Bad A, Serna Ríos C. *Censo General 2005* Departamento Administrativo Nacional de Estadística (DANE) (2006).
